# Global gene expression analysis of systemic sclerosis myofibroblasts demonstrates a marked increase in the expression of multiple NBPF genes

**DOI:** 10.1038/s41598-021-99292-y

**Published:** 2021-10-14

**Authors:** Giuseppina Abignano, Heidi Hermes, Sonsoles Piera-Velazquez, Sankar Addya, Francesco Del Galdo, Sergio A. Jimenez

**Affiliations:** 1 Leeds Institute of Rheumatic and Musculoskeletal Medicine, University of Leeds, Beckett Street, WTBB 6.14, Leeds, LS97TF UK; 2 NIHR Leeds Biomedical Research Centre, Leeds Teaching Hospitals NHS Trust, Leeds, UK; 3 Jefferson Institute of Molecular Medicine and Scleroderma Center, Thomas Jefferson University, 233 S. 10th Street, Room 509 BLSB, Philadelphia, PA 19107-5541 USA; 4 Center for Genomics, Kimmel Cancer Center, Thomas Jefferson University, Philadelphia, PA 19107-5541 USA

**Keywords:** Systemic sclerosis, Microarray analysis, Reverse transcription polymerase chain reaction

## Abstract

Myofibroblasts are the key effector cells responsible for the exaggerated tissue fibrosis in Systemic Sclerosis (SSc). Despite their importance to SSc pathogenesis, the specific transcriptome of SSc myofibroblasts has not been described. The purpose of this study was to identify transcriptome differences between SSc myofibroblasts and non-myofibroblastic cells. Alpha smooth muscle actin (α-SMA) expressing myofibroblasts and α-SMA negative cells were isolated employing laser capture microdissection from dermal cell cultures from four patients with diffuse SSc of recent onset. Total mRNA was extracted from both cell populations, amplified and analyzed employing microarrays. Results for specific genes were validated by Western blots and by immunohistochemistry. Transcriptome analysis revealed 97 differentially expressed transcripts in SSc myofibroblasts compared with non-myofibroblasts. Annotation clustering of the SSc myofibroblast-specific transcripts failed to show a TGF-β signature. The most represented transcripts corresponded to several different genes from the Neuroblastoma Breakpoint Family (NBPF) of genes. NBPF genes are highly expanded in humans but are not present in murine or rat genomes. In vitro studies employing cultured SSc dermal fibroblasts and immunohistochemistry of affected SSc skin confirmed increased NBPF expression in SSc. These results indicate that SSc myofibroblasts represent a unique cell lineage expressing a specific transcriptome that includes very high levels of transcripts corresponding to numerous NBPF genes. Elevated expression of NBPF genes in SSc myofibroblasts suggests that NBPF gene products may play a role in SSc pathogenesis and may represent a novel therapeutic target.

## Introduction

Systemic Sclerosis (SSc) is a systemic autoimmune disease of unknown etiology characterized by progressive fibrosis of skin and multiple internal organs and a severe proliferative vasculopathy^1–4^. The cells responsible for tissue fibrosis and fibroproliferative processes are myofibroblasts, a population of mesenchymal cells displaying unique biological functions including cell mobility and tissue contraction, and the high expression of α-smooth muscle actin (α-SMA) and numerous molecules associated with fibrotic processes^5–8^. The accumulation of myofibroblasts in affected tissues and their expression of a persistent profibrotic phenotype are crucial in the development of the fibroproliferative process in SSc. There is also strong evidence to indicate that these cells regulate the extent and rate of progression of the fibrotic alterations in SSc, and, therefore, play an important role in determining the clinical course, response to therapy, prognosis, and overall mortality of the disease^9–13^. Indeed, it has been shown that the number of myofibroblasts present in affected SSc skin correlates with the severity of SSc-associated tissue fibrosis^12^.

The origins of the myofibroblasts responsible for the exaggerated and uncontrolled production of collagen and other extracellular matrix proteins in SSc have not been completely elucidated. Extensive studies have shown that these cells may originate from several sources, including expansion and activation of resident tissue fibroblasts^13^, migration and tissue accumulation of bone marrow-derived circulating fibrocytes^14,15^, or from trans-differentiation of epithelial or endothelial cells to mesenchymal cells, complex processes known, respectively, as epithelial to mesenchymal transition^16–19^ and endothelial to mesenchymal transition^20–23^. The relative contribution and importance of the different myofibroblast sources to the fibrotic alterations in SSc is unknown, and it is likely to depend on the stage of evolution of the disease as well as on the specific organ involved. Furthermore, it is likely that myofibroblasts originated from different cellular sources or present in different tissues may display unique features or play distinct roles in the pathogenesis or perpetuation of tissue fibrosis in SSc^7–13,24^. For example, it has been shown that the global gene expression profile of SSc myofibroblasts present in lungs from patients with SSc-associated interstitial lung disease examined employing single cell RNA sequencing assays displays unique gene expression signatures compared to two different types of fibroblasts^25^. However, the gene expression profile of SSc dermal myofibroblasts has not been examined and it is not known whether these cells may represent a specific cellular phenotype. Thus, the purpose of this study was to analyze the specific transcriptome of pure SSc myofibroblasts. To accomplish this goal myofibroblasts expressing α-SMA actin were isolated employing laser capture microdissection (LCM) from monolayer cultures of cells derived from skin biopsies from affected SSc skin.

## Results

### Laser capture microdissection of α-SMA-positive myofibroblasts and α-SMA-negative fibroblasts

**Figure 1:**
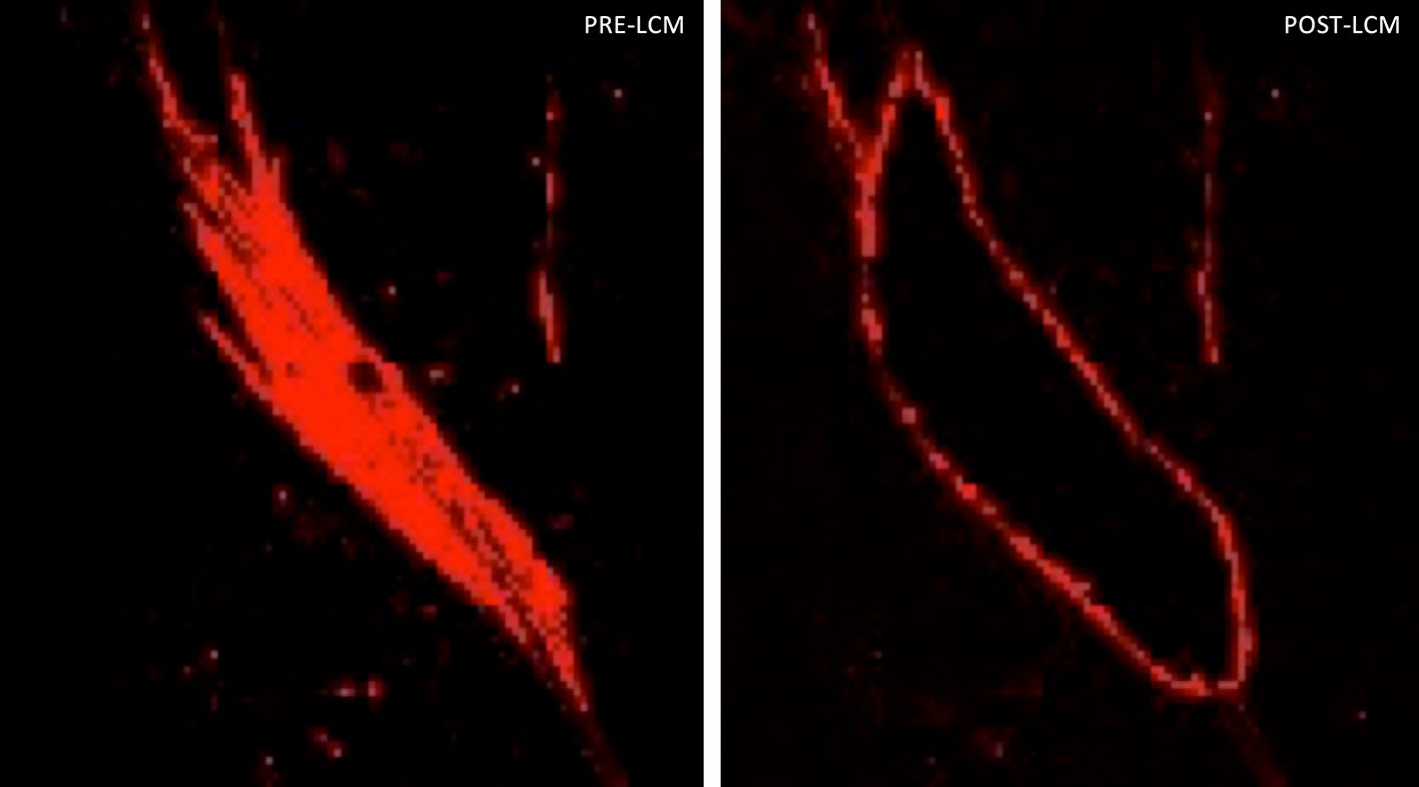
Laser capture microdissection of SSc myofibroblasts from monolayer cultures of dermal fibroblasts expanded from affected SSc skin in vitro. The image shows immunofluorescence staining (red) for α-SMA before (PRE-LCM) and the empty space following its removal (POST-LCM) employing laser capture microdissection. The image is representative of 250 cells per experiment and four different experiments from cultures obtained from four different dcSSc skin biopsies. Original magnification 400 × .

To determine the global transcriptome of α-SMA positive SSc myofibroblasts we examined monolayer cell cultures that had been expanded from biopsies excised from the leading (proximal) edge of forearm skin lesions of four patients with dcSSc of recent onset. Passage 3 cells were dissociated and subcultured directly on Leika LCM slides and when the slides were essentially fully covered by growing cells, a quick fixation/staining protocol on ice was performed to preserve the mRNA integrity. Alpha-SMA positive myofibroblasts and α-SMA-negative fibroblasts were isolated employing LCM (Fig. 1) and the mRNA of the separate populations was amplified. Real Time PCR was used to confirm the enrichment of α-SMA in the myofibroblasts. The results showed that α-SMA expression was an average of 370% greater in the α-SMA-positive myofibroblasts compared to the α-SMA-negative fibroblasts as described elsewhere^26^.

### Differential gene expression of α-SMA-positive SSc myofibroblasts compared with α-SMA-negative SSc fibroblasts

**Figure 2:**
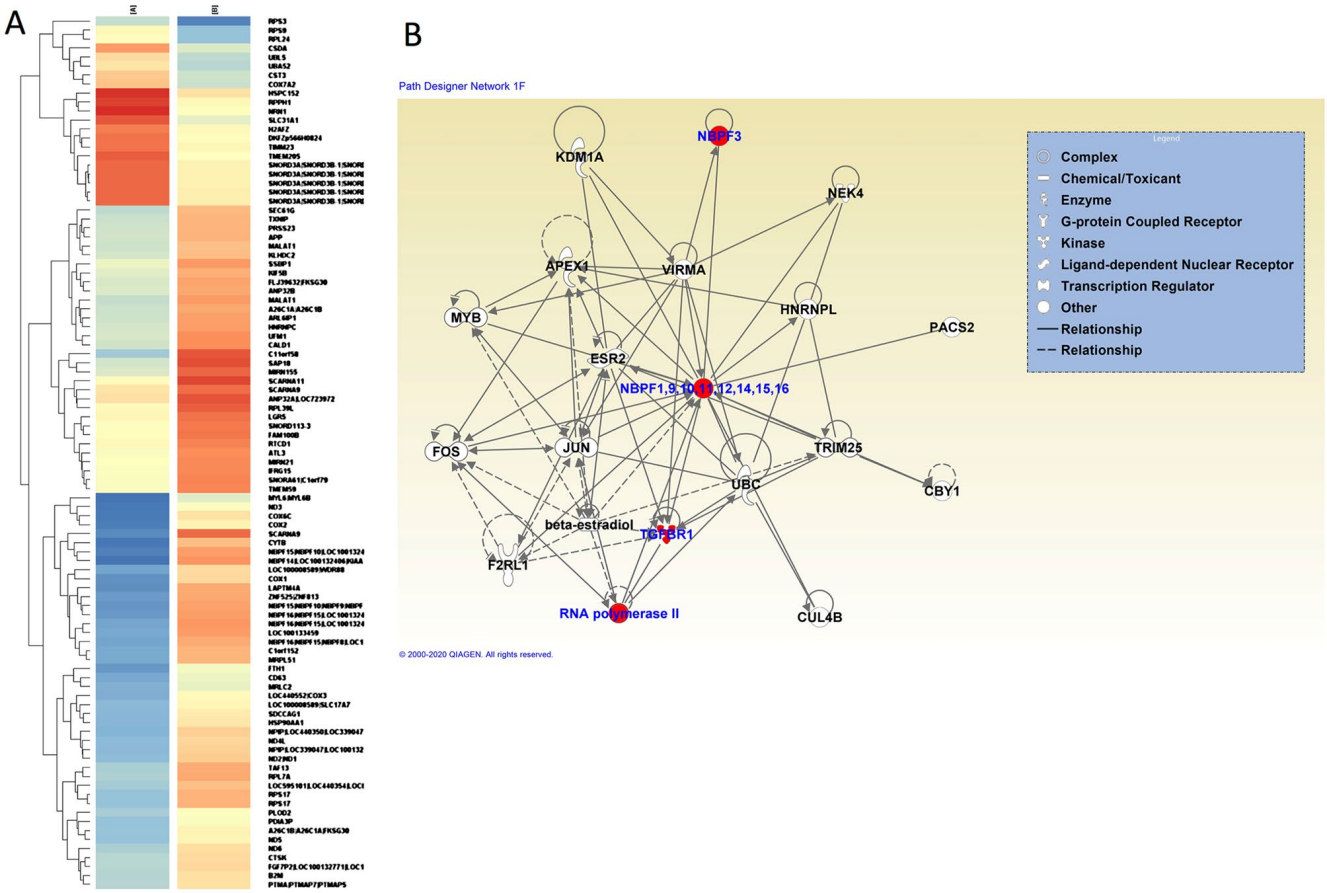
Hierarchic clustering of the gene expression levels in SSc myofibroblasts compared to non-activated fibroblasts. Comparison of gene expression levels of α-SMA positive SSc myofibroblasts and α-SMA negative SSc fibroblasts isolated from the same culture dish employing LCM. (**A**) The image shows the heat map containing the genes as an average of the two sets of samples (4 α-SMA-negative “A” samples vs. 4 α-SMA-positive “B” samples). Gene expression levels are depicted as color variation from red (high expression) to blue (low expression). The gene expression microarrays were performed employing Affymetrix gene chips (human gene 1.0 ST arrays), and the analysis were performed utilizing the Agilent Gene Spring Software 11.5 also from Affymetrix. (**B**) Schematic representation of the genes interacting with NBPF genes according to Ingenuity Pathway Analysis (IPA), performed to obtain biological and functional network analysis and utilizing ingenuity.com 8.0 software. The complete list of the genes and pathways identified by Ingenuity CanPath is shown in Supplementary Table S1.

The global mRNA expression pattern of α-SMA-positive SSc myofibroblasts isolated employing LCM from monolayer cell cultures derived from affected SSc skin identified 97 genes differentially expressed (greater than 1.5 fold with a *p* value of < 0.5) compared to the transcriptome of LCM captured α-SMA-negative SSc fibroblasts. Twenty one transcripts were downregulated and 76 upregulated (Supplementary Data S1). A representative heat map of the corresponding genes as an average of the two sets of samples (4 α-SMA-negative samples vs. 4 α-SMA-positive samples) is shown in Fig. 2A. A heat map showing the 8 samples in one single figure, and 4 additional heat maps showing separate images of each individual pair comparison are included in Supplementary Figs. S1 and S2. The full microarray data are publicly available through NCBI GEO website (http://www.ncbi.nlm.nih.gov/geo/). Annotation Clustering analysis indicated that the transcripts with the highest enrichment score corresponded to members of the NBPF cluster which displayed an enrichment score of 2.93. Multiple NBPF transcripts were found in the array, that corresponded to at least six different NBPF genes (Fig. 2A). Canonical pathway analysis according to Ingenuity software identified numerous relevant genes related to inflammatory and fibrotic pathways. A diagram of the genes interacting with NBPF genes is shown in Fig. 2B. A full list of CanPath analysis is summarized in Supplementary Table S1. Data of the quality and quantity of RNA samples used in the microarray experiments are shown in Supplementary Fig. S4 reporting the list of the RNA samples with their concentrations, the 260/280 ratio for demonstration of their high purity and the nanodrop analysis.

### NBPF protein expression in systemic sclerosis affected skin

**Figure 3:**
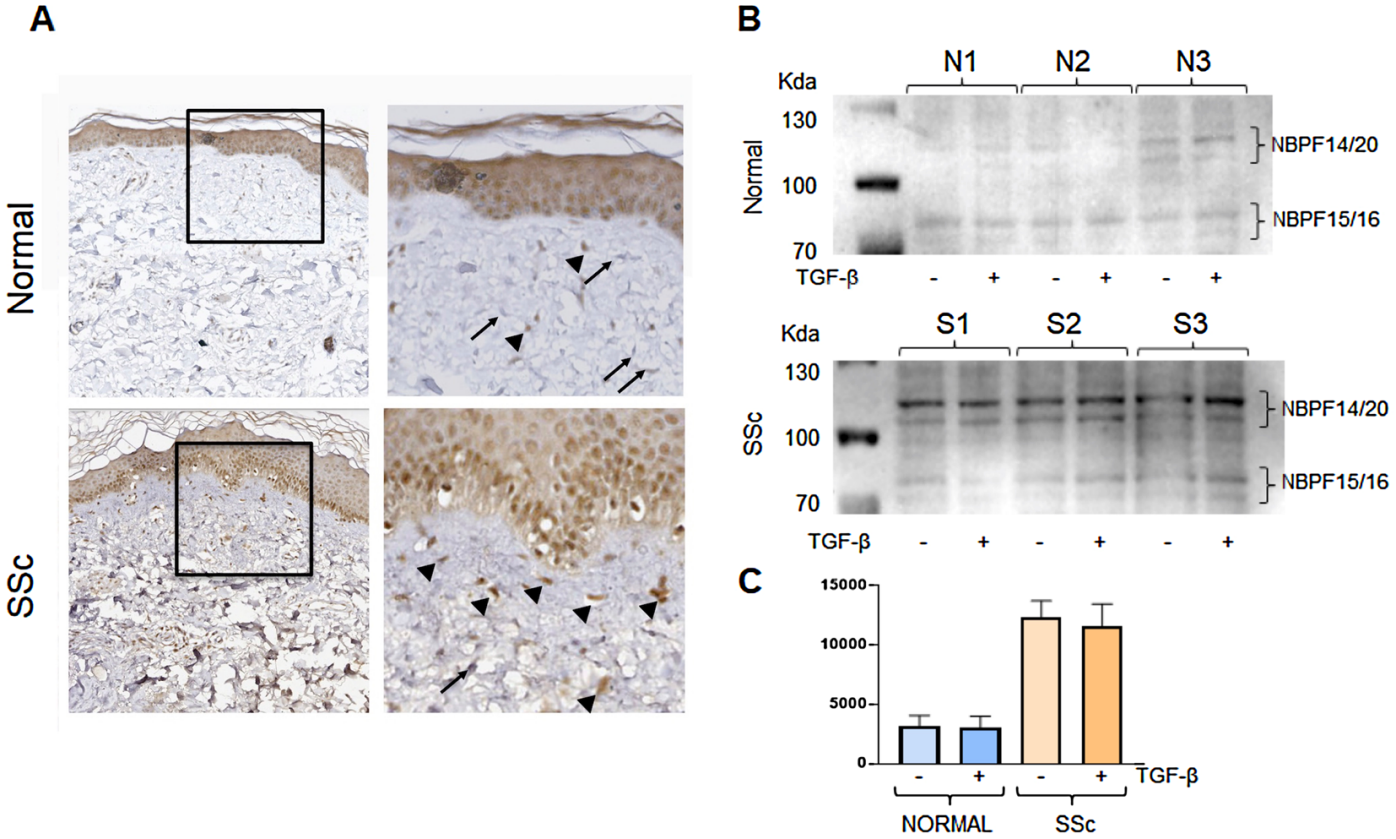
Increased expression of NBPF in affected SSc skin and in cultured SSc dermal fibroblasts. (**A**) Immunohistochemistry for NBPF on one representative skin biopsy from a healthy control (normal) or from a SSc patient. Note that fibroblasts within healthy control skin did not show any staining for NBPF (black arrows) whereas fibroblasts within SSc dermis were intensely stained in brown (arrow heads). Original magnification 20X. Panels are representative of five independent skin biopsies. Panels on the right are a tenfold magnification of the squared inset in the panels on the left. (**B**) Western blots of cell extracts from three different normal fibroblast cell lines and three different SSc dermal fibroblast cell lines cultured in monolayers with or without TGF-β1 (10 ng/ml) for 24 h employing an NBPF antibody. The NBPF bands shown have been identified based on their expected molecular weights. (**C**) Bar graph displaying a quantitative assessment of the data shown in (**B**). The gel blots used to prepare the images shown in (**B**) and (**C**) were cropped above the 130 KD and below the 70 KD molecular size marker bands because the migration of the relevant NBPF molecular species was expected to be between these molecular sizes. There were no other crops in the gel blots. Full blots of Western Blot included in this figure are shown in Supplementary Fig. S5.

To determine whether the increased expression of NBPF observed in the microarray of the LCM SSc myofibroblasts was also present in vivo, we analysed by immunohistochemistry skin biopsies from three dcSSc patients and biopsies from three healthy subjects obtained from the same anatomical region (forearm). Immunohistochemistry studies indicated that epithelial cells within the epidermis showed a strong and homogeneous NBPF staining in both healthy control skin and in SSc skin biopsies. In contrast, in SSc skin biopsies virtually all the cellular elements within the papillary dermis, showed a strong positivity for NBPF whereas in the dermis of healthy control skin NBPF expression was confined to fewer cells (Fig. 3A). These observations were confirmed by Western Blot analysis of cell extracts from confluent cultures of dermal fibroblasts established from patients with dcSSc and from normal control skin. These studies showed a substantial increase in protein bands corresponding to numerous NBPF transcript levels in cultures of SSc dermal fibroblasts compared to normal dermal fibroblasts (Fig. 3B, C).

### Dermal fibroblast expression of NBPF in vitro is not stimulated by TGF-β

To examine whether the expression levels of NBPF gene products were modified by exposure to TGF-β, qRT-PCR and Western blot analysis of normal dermal fibroblasts cultured in vitro in the presence of TGF-β were examined. The results showed that incubation of the cells with 10 ng/ml of human recombinant TGF-β for either 24 or 48 h failed to induce any detectable increase in the expression of NBPF at either the mRNA or the protein level (data not shown). Similar results were obtained at the protein level following treatment of cultured normal and SSc dermal fibroblasts with TGF-β for 24 h as shown in Fig. 3B, C.

## Discussion

Extensive experimental evidence indicates that myofibroblasts are the key cellular elements in normal tissue repair responses^5–8^ and have been shown to play a crucial role in the pathogenesis of tissue fibrosis in SSc and other fibrotic disorders^9–12^. It has already been shown that these cells may originate from numerous sources^7,13,24^, including TGF-β-induced activation of quiescent fibroblasts^7,8,24^ as well as from circulating fibrocytes^14,15^ or from epithelial or endothelial cells undergoing a phenotypic transition into mesenchymal cells^16–23^. However, there is extreme heterogeneity in their proportion compared to non-myofibroblasts in the skin of SSc patients^9–13^. The unequivocal evidence that within affected SSc skin there is remarkable heterogeneity and variability in the abundance of myofibroblasts compared to that of quiescent fibroblasts is of remarkable interest taking into account the premise that essentially all cells within a skin biopsy are exposed to the same microenvironment in vivo.

Our study aimed to identify the differences in gene expression between α-SMA-positive LCM isolated myofibroblasts and LCM isolated α-SMA-negative fibroblasts derived from monolayer cultures established from skin biopsies from patients with diffuse SSc of resent onset. Microarray analysis indicated that 97 transcripts were differentially expressed in the myofibroblasts compared to the non-myofibroblasts isolated employing LCM from the same monolayer cultures of SSc skin derived cells. Among these, some transcripts were clearly related to profibrotic activation, whereas others were ribosomal genes, mitochondrial genes involved in oxidative phosphorylation, or genes involved in cell to cell adhesion and in antigen processing and presentation. A remarkable observation was that the most enriched family of genes within the LCM SSc myofibroblast signature was the NBPF family, which was represented by transcripts corresponding to at least 6 different NBPF genes. This observation is of substantial interest owing to the fact that the NBPF genes are highly enriched in humans as a result of a marked genetic expansion and human lineage-specific amplification and selection that occurred in the human genome following the evolutionary separation of Homo sapiens from chimpanzees^27,28^. The NBPF encoded genes represent a large gene family comprising as many as 25 distinct genes that are localized almost exclusively within chromosome 1. This large gene family received the nomenclature of NBPF genes because one of its members (NBPF1) was found to be disrupted by a chromosomal translocation in a neuroblastoma patient. Although, at the present, the function(s) of NBPF gene products have not been fully elucidated it has become apparent that besides a possible role in the development of neuroblastoma, the proteins encoded by these genes have important functions in brain development and cognitive functions^29^. However, the full functional range of these genes is not known. Indeed, it has been recently demonstrated that NBPF encoded proteins may participate in inflammatory/immunologic reactions owing to their role as putative transcription factors responsive to NFkB^30^. While the lack of functional data on NBPF prevents to imply a specific role in SSc pathogenesis, the remarkable increase in the expression of transcripts corresponding to several NBPF genes in myofibroblasts obtained from SSc patients supports the possibility that these highly expanded human lineage genes could be involved in the pathogenesis of a disease such as SSc that displays human-specific expression^31,32^. On the other hand, it is possible that NBPF is regulated by matrix stiffness since all the experiments were performed under regular culture conditions, which corresponds to a stiff matrix. Numerous recent studies have indicated a highly important role of matrix stiffness and of the biophysical properties of the extracellular matrix in the regulation of gene expression including a marked profibrotic effect of a stiff matrix on fibroblast/myofibroblast biosynthetic patterns^33,34^. This aspect is of high relevance and will need to be examined in greater detail. However, it should be emphasized that since both types of laser captured cells studied here were obtained from the same culture matrix it is unlikely that matrix stiffness played a role in the markedly increased differential expression of NBPF gene transcripts we observed.

It is generally accepted that TGF-β plays a crucial role in the pathogenesis of SSc- associated fibrosis^35–38^. Furthermore, it has been shown in numerous in vitro studies that TGF-β is capable of inducing a potent stimulation of the conversion of quiescent mesenchymal fibroblastic cells to activated myofibroblasts^39^. However, a recent study that examined regulators involved in the phenotype of cultured primary SSc dermal fibroblasts employing RNA interference failed to demonstrate a role for TGF-β in the induction of SSc myofibroblasts^40^. The data presented here are in agreement with these latter observations.

Limitations of our study included the small number of enrolled patients and controls and the lack of correction for multiple comparison, however the results of this study clearly indicate that SSc myofibroblasts may represent a specific lineage of mesenchymal cells with a specific transcriptional signature that could determine a functional phenotype playing a crucial role in the pathogenesis of the SSc-related fibrotic process. The identification of a specific myofibroblast cellular phenotype in SSc with a unique gene expression profile quite distinct from that induced by TGF-β could pave the way to a more precise understanding of the complex pathogenesis of tissue fibrosis in SSc and may be of great value for the development of novel therapies for this currently incurable disease.

## Methods

### Patients and skin biopsies

Full thickness skin biopsies were surgically obtained from the forearms of four adult patients with SSc of recent onset, which was defined as disease duration of less than 18 months from the appearance of clinically detectable skin induration. All participants provided written informed consent to participate in this study. Informed consent procedure and all experiments were approved by the Institutional Review Board of Thomas Jefferson University (IRB#: 06F.186). All experiments were performed in accordance with relevant guidelines and regulations. The patients satisfied the 2013 ACR/EULAR criteria for the classification of SSc^41^ and had the diffuse cutaneous clinical subset (dcSSc) as defined by LeRoy et al.^42^. The SSc skin biopsies were obtained from the proximal edge of expanding forearm lesions. One portion of the biopsies was fixed in formaldehyde and embedded in paraffin for diagnostic and other histopathological and immunohistochemical studies. The remaining tissues were used for isolation and establishment of monolayer cultures of dermal cell lines as described previously^43,44^. All participants provided written informed consent to participate in this study. Informed consent procedure was approved by the Institutional Review Board of Thomas Jefferson University (IRB#: 06F.186) as described previously^43,44^.

### Cell cultures

Cell cultures were established from SSc skin biopsies obtained from the leading edge of forearm lesions from untreated patients with dcSSc of recent onset and from the dorsal side of forearms from normal volunteers as described previously^43,44^. The cells were cultured in Dulbecco’s Modified Eagle’s Medium (DMEM) (Invitrogen, Carlsbad, CA) supplemented with 10% FBS, antibiotics and glutamine (complete medium) until confluent and were used before reaching passage 6.

### Laser capture microdissection (LCM)

For LCM, third passage monolayer cultures of dermal cells derived from the skin biopsies of four SSc patients were dissociated and plated directly onto polyethylene naphthalate membrane-coated slides (Leica Microsystems, Wetzlar, Germany), which had been pretreated with RNAse Zap (Ambion, Life Technologies, NY), sterilized with 100% ethanol, and allowed to air dry under an ultraviolet light prior to being placed in a standard polystyrene tissue culture dish. Following 48 h culture, the cells were fixed with acetone and incubated for 7 min on dry ice with a mouse anti-α-SMA primary antibody (1:50, Thermo Fisher Scientific, Fremont, CA), followed by 3 min incubation with fluorescent rabbit anti-mouse Cy3 secondary antibody (1:200, Sigma-Aldrich, Saint Louis, MO). The antibody diluent consisted of 2% BSA in PBS with added RNAse inhibitor (1 U/µl final concentration). Cold PBS was used for all interval rinses. The slides were subsequently dehydrated in serial ethanol dilutions and placed on dry ice. LCM and pressure cell capturing were performed immediately following antibody staining according to a modification of previously published procedures^45^. The laser capture microdissected cells were collected in RNA-ase free tubes to avoid RNA degradation, and were stored at − 80 °C until processed as described in detail previously^26^.

### Immunohistochemistry

For Immunohistochemistry, paraffin-embedded skin sections from 3 dcSSc patients and 3 sex- and age-matched healthy controls, were incubated with heat retrieval solution (Access Revelation, Menapath, A. Menarini Diagnostics, UK) and then transferred to the slide wash following heat mediated antigen retrieval according to the manufacturer’s protocol. Slides were then incubated with 3% H_2_O_2_ for 20 min to inhibit endogenous peroxidase activity and for additional 20 min with 1% casein, for protein-blocking. Neuroblastoma Breakpoint Family (NBPF) was detected by incubation at a 1:50 dilution, for 2 h at room temperature with a polyclonal anti-NBPF antibody that detects endogenous levels of total NBPF including NBPFs 1/9/10/12/14/15/16 and 20 (NBPF1-71614; Novus Biologicals, Littleton, CO). Slides were then incubated with NovocastraTM Post Primary (Leica Biosystems, UK) for 30 min, washed in TBS and incubated with NovolinkTM Polymer for 30 min to detect the tissue–bound primary antibody. Sections were further incubated with the substrate/chromogen, 3,3’–diaminobenzidine (DAB), prepared from NovocastraTM DAB Chromogen and NovolinkTM DAB substrate buffer, according to the manufacturer’s protocol. Sections were counterstained with hematoxylin.

### RNA isolation, amplification and quantitative real time PCR

Two hundred and fifty α-SMA positive SSc myofibroblasts and two hundred and fifty α-SMA negative SSc fibroblasts were harvested by LCM as described above and then processed for RNA extraction (RNAeasy kit; Qiagen, Valencia, CA) as described previously^26,46^. Normal and SSc human dermal fibroblasts were cultured in monolayers until reaching confluency and then they were treated with trypsin–EDTA, washed in PBS and then processed for RNA extraction (RNAeasy kit; Qiagen, Valencia, CA) according to the manufacturer’s instructions. Amplified cDNA was prepared using nuGEN WT-Ovation RNA amplification system (nuGEN, San Carlos, CA) followed by the NuGEN WT-exon module. Total RNA (2 µg) was reverse-transcribed using Super-script-II reverse transcriptase (Invitrogen, Carlsbad, CA). Real-time PCR was performed using SYBR green Master mix chemistry employing a standard amplification protocol on a Bio- Rad MyiQ real-time PCR system (Bio-Rad, Hercules, CA). Reactions were conducted as described previously^26,46^. All primers were designed using Primer Express (Applied Biosystems, Foster City, CA) and validated employing the National Center for Biotechnology Information (NCBI) database for specificity. The primers used to amplify NBPF sequences were: Forward-TAAGGGAGAAGTTGCGGGAA; and Reverse-AGTGAGGAGGGCCTGGAGAT.

### Global gene expression profiling of LCM-isolated SSc myofibroblasts

Total RNA was extracted from LCM isolated SSc myofibroblasts and non-myofibroblastic SSc cells using the Qiagen RNeasy Mini kit (Qiagen, Valencia, CA). A DNase I digestion step was included to eliminate DNA contamination. Four separate pairs of samples were studied. Assessment for each group was performed in triplicate. RNA was quantified on a NanoDrop spectrophotometer (Nanodrop- ThermoSACientific, Wilmington. DE), followed by RNA quality assessment on an Agilent 2100 bioanalyzer (Agilent, Palo Alto, CA, USA). Amplification of cDNA was performed using the Ovation Pico WTA-system V2 RNA amplification system (NuGen Technologies, Inc.). Briefly, 50 ng total RNA was reverse transcribed using a chimeric cDNA/mRNA primer, and a second complementary cDNA strand was synthesized. Purified cDNA was then amplified with ribo-SPIA enzyme and SPIA DNA/RNA primers (NuGEN Technologies, Inc.). Amplified DNA was purified with Qiagen MinElute (Qiagen) reaction cleanup kit. The concentration of purified cDNA was measured using the NanoDrop spectrophotometer and 2.5 µg cDNAs were fragmented and chemically labeled with biotin to generate biotinylated ST-cDNA using FL-Ovation cDNA biotin module V2 (NuGen Technologies, Inc.).

Global gene expression microarrays were performed employing Affymetrix gene chips (human gene 1.0 ST array; Affymetrix, Santa Clara, CA) as described previously^26,46^. The gene chips were hybridized with fragmented and biotin-labeled target (2.5 µg) in 110 µl of hybridization cocktail. Target denaturation was performed at 99 °C for 2 min and then 45 °C for 5 min followed by hybridization for 18 h. Arrays were then washed and stained using Gene chip Fluidic Station 450, using Affimetrix Gene chip hybridization wash and stain kit. Chips were scanned on an Affymetrix Gene Chip Scanner 3000, using Command Console Software. Data analyses were performed using Gene Spring software 11.5 (Agilent Technologies, Inc., Santa Clara, CA). The probe set signals were calculated with the Iterative Plier 16 normalization algorithm; baseline to median of all samples was used as baseline option. The data were filtered by percentile and a lower cut off was set at 25. The criteria for differentially expressed genes were set at ≥ 1.5-fold changes. Statistical analysis was performed to compare the results obtained from 4 samples of α-SMA-positive SSc myofibroblasts displaying the highest α-SMA transcript levels to the results obtained from 4 samples of α-SMA-negative SSc fibroblasts using T-Test unpaired with *p*-value equal or less than 0.5. Heat maps were generated from the differentially expressed gene list. The list of differentially expressed genes was loaded into Ingenuity Pathway Analysis (IPA) 8.0 software (http://www.ingenuity.com) to perform biological network and functional analyses.

### Western blot analysis

Dermal fibroblasts isolated from SSc patients and healthy donors were cultured in monolayers with and without TGF-β1 (10 ng/mL) for 24 h, then harvested and lysed in RIPA lysis buffer containing protease inhibitors. For Western blots, aliquots of the extracts containing 30 µg of protein were separated by electrophoresis in 8% TrisGlycine polyacrylamide gels and the separated proteins were electroblotted onto nitrocellulose membranes (Invitrogen). The membranes were blocked in PBS/0.5% dry milk/0.01% normal goat serum for 1 h at room temperature. The transferred proteins were incubated overnight at 4 °C with an NBPF antibody that recognizes NBPF species 1, 9, 10, 12, 14, 15, 16 and 20 (Assay Biotech, Fremont CA). Rabbit secondary antibody coupled to peroxidase and the ECL detection system (Thermo Scientific Pierce) were employed for detection. Quantitative assessment of the protein in the blots was performed employing quantitative densitometry. The identity of the various NBPF protein bands was assigned based on their estimated molecular weight.

## Supplementary Information

Supplementary Information 1.

Supplementary Information 2.

## Data Availability

The full microarray data are publicly available through NCBI GEO website (http://www.ncbi.nlm.nih.gov/geo/).
