# Supplementary material for: Global gene expression analysis of systemic sclerosis myofibroblasts demonstrates a marked increase in the expression of multiple NBPF genes
Source: Sci Rep. 2021 Oct 14;11:20435. doi: 10.1038/s41598-021-99292-y (PMC8516909; doi:10.1038/s41598-021-99292-y)

Supplementary File

Global gene expression analysis of Systemic Sclerosis myofibroblasts demonstrates a marked increase in the expression of multiple NBPF genes

Authors: Giuseppina Abignano[#1,2], Heidi Hermes[#3], Sonsoles Piera-Velazquez[3], Sankar Addya[4], Francesco Del Galdo[^1,2*], and Sergio A. Jimenez[^3*].

1. Leeds Institute of Rheumatic and Musculoskeletal Medicine, University of Leeds, United Kingdom.
2. NIHR Leeds Biomedical Research Centre, Leeds Teaching Hospitals NHS Trust, Leeds, United Kingdom.
3. Jefferson Institute of Molecular Medicine and Scleroderma Center, Thomas Jefferson University, USA.
4. Center for Genomics, Kimmel Cancer Center, Thomas Jefferson University, USA.

# These two authors contributed equally

^ These two authors contributed equally

*These two authors are both corresponding authors.

*Correspondence: f.delgaldo@leeds.ac.uk; sergio.jimenez@jefferson.edu

Supplementary Figure S1. Heat map showing gene expression levels of the eight samples [4 α-SMA negative (“A” samples) and 4 α-SMA positive (“B” samples) SSc fibroblasts] isolated from the same culture dish employing LCM. Gene expression levels are depicted as color variation from red (high expression) to blue (low expression). The gene expression microarrays were performed employing Affymetrix gene chips (human gene 1.0 ST arrays), and the analysis were performed utilizing the Agilent Gene Spring Software 11.5 also from Affymetrix.

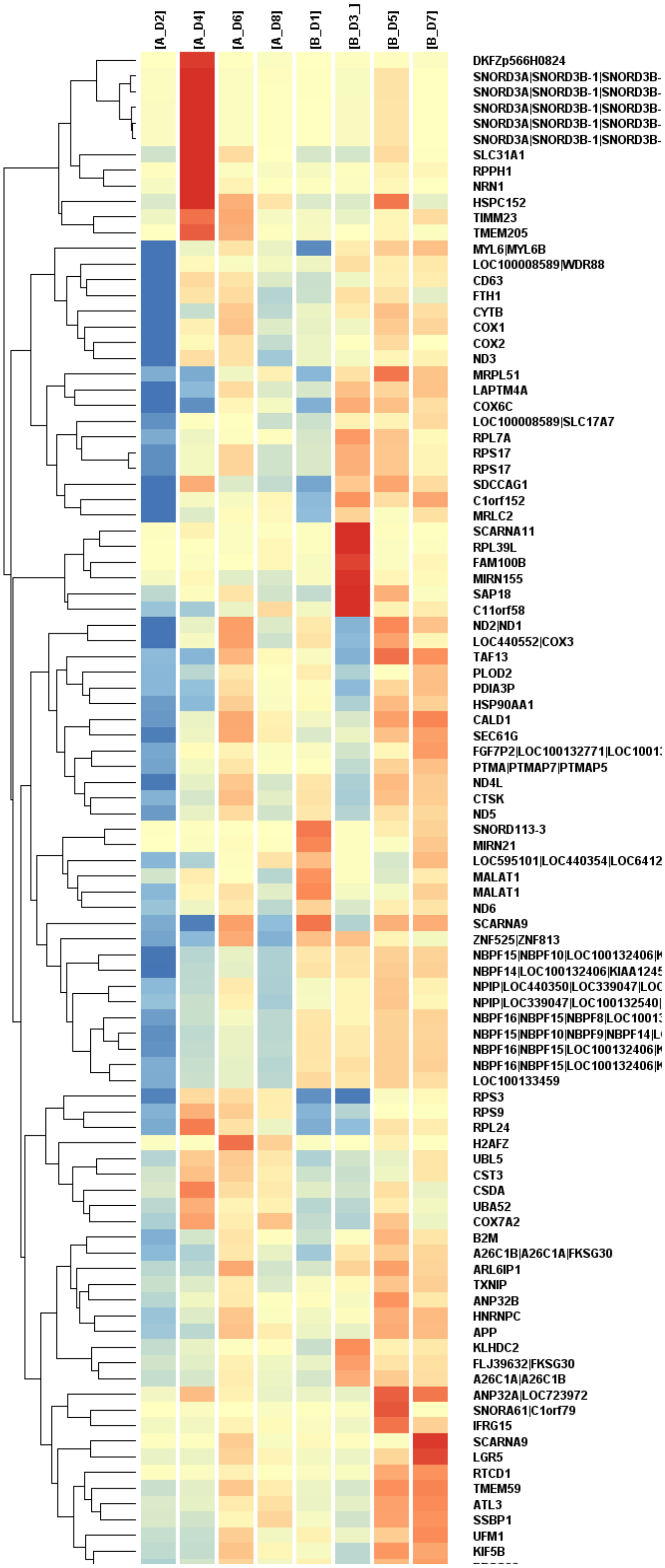

Supplementary Figure S2. Four heat maps showing gene expression level as separate images of each individual pair (α-SMA-negative "A" vs. α-SMA-positive "B" samples). The gene expression microarrays were performed employing Affymetrix gene chips (human gene 1.0 ST arrays), and the analysis were performed utilizing the Agilent Gene Spring Software 11.5 also from Affymetrix.

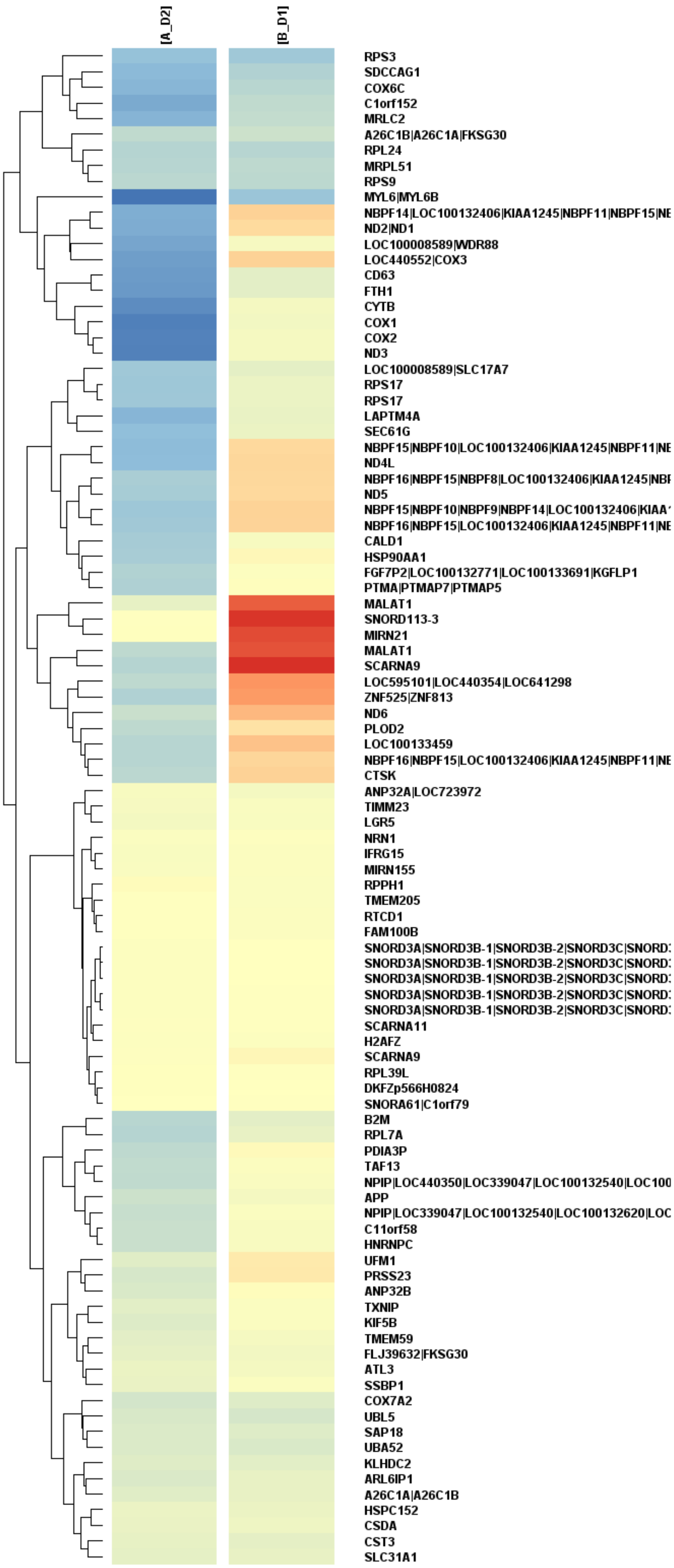

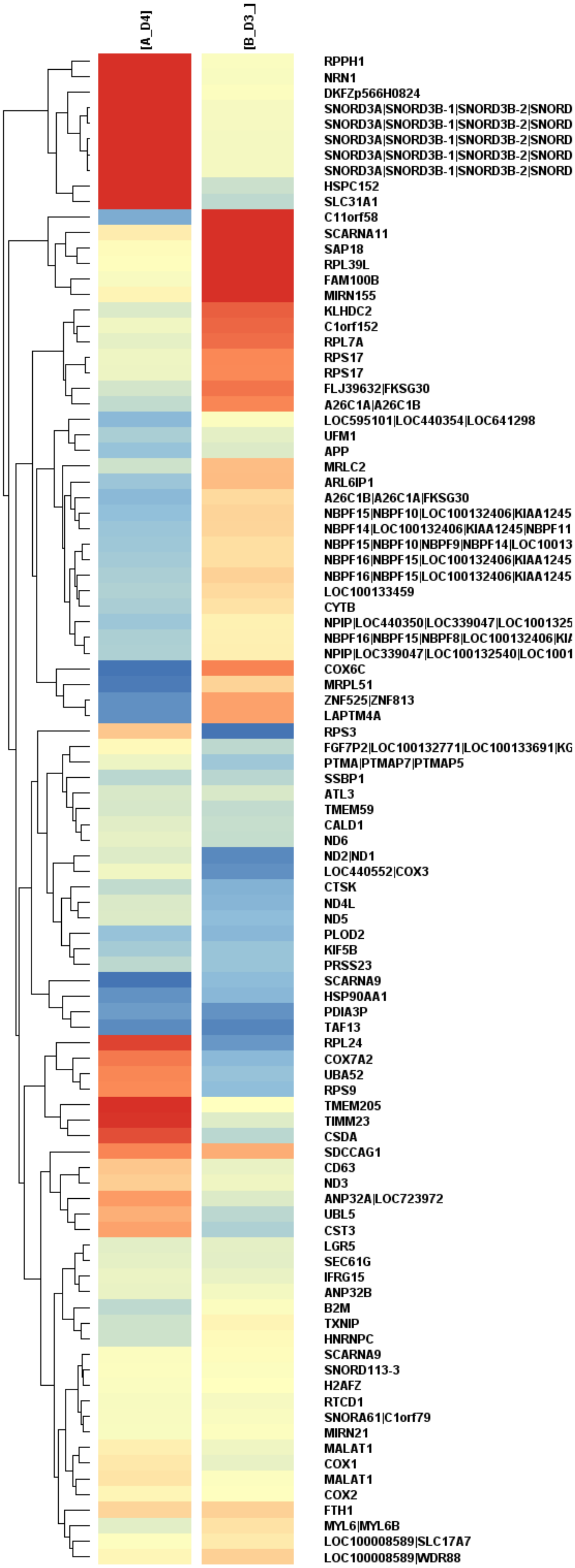
[A_D4]
[B_D3_]
RPPH1
NRN1
DKFZp566H0824
SNORD3A|SNORD3B-1|SNORD3B-2|SNORD
SNORD3A|SNORD3B-1|SNORD3B-2|SNORD
SNORD3A|SNORD3B-1|SNORD3B-2|SNORD
SNORD3A|SNORD3B-1|SNORD3B-2|SNORD
SNORD3A|SNORD3B-1|SNORD3B-2|SNORD
HSPC152
SLC31A1
C11orf58
SCARNA11
SAP18
RPL39L
FAM100B
MIRN155
KLHDC2
C1orf152
RPL7A
RPS17
RPS17
FLJ39632|FKSG30
A26C1A|A26C1B
LOC595101|LOC440354|LOC641298
UFM1
APP
MRLC2
ARL6IP1
A26C1B|A26C1A|FKSG30
NBPF15|NBPF10|LOC100132406|KIAA1245
NBPF14|LOC100132406|KIAA1245|NBPF11
NBPF15|NBPF10|NBPF9|NBPF14|LOC10013
NBPF16|NBPF15|LOC100132406|KIAA1245
NBPF16|NBPF15|LOC100132406|KIAA1245
LOC100133459
CYTB
NPIP|LOC440350|LOC339047|LOC1001325
NBPF16|NBPF15|NBPF8|LOC100132406|KIA
NPIP|LOC339047|LOC100132540|LOC1001
COX6C
MRPL51
ZNF525|ZNF813
LAPTM4A
RPS3
FGF7P2|LOC100132771|LOC100133691|KG
PTMA|PTMAP7|PTMAP5
SSBP1
ATL3
TMEM59
CALD1
ND6
ND2|ND1
LOC440552|COX3
CTSK
ND4L
ND5
PLOD2
KIF5B
PRSS23
SCARNA9
HSP90AA1
PDIA3P
TAF13
RPL24
COX7A2
UBA52
RPS9
TMEM205
TIMM23
CSDA
SDCCAG1
CD63
ND3
ANP32A|LOC723972
UBL5
CST3
LGR5
SEC61G
IFRG15
ANP32B
B2M
TXNIP
HNRNPC
SCARNA9
SNORD113-3
H2AFZ
RTCD1
SNORA61|C1orf79
MIRN21
MALAT1
COX1
MALAT1
COX2
FTH1
MYL6|MYL6B
LOC100008589|SLC17A7
LOC100008589|WDR88

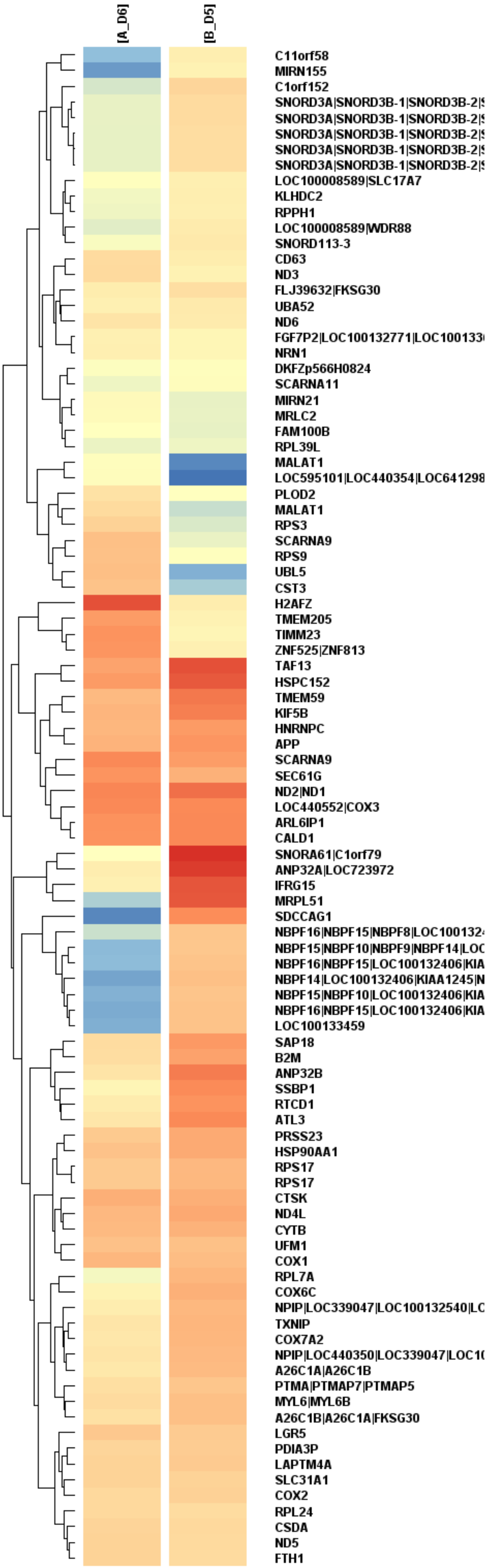
[A_D6]
[B_D5]
C11orf58
MIRN155
C1orf152
SNORD3A|SNORD3B-1|SNORD3B-2|
SNORD3A|SNORD3B-1|SNORD3B-2|
SNORD3A|SNORD3B-1|SNORD3B-2|
SNORD3A|SNORD3B-1|SNORD3B-2|
SNORD3A|SNORD3B-1|SNORD3B-2|
LOC100008589|SLC17A7
KLHDC2
RPPH1
LOC100008589|WDR88
SNORD113-3
CD63
ND3
FLJ39632|FKSG30
UBA52
ND6
FGF7P2|LOC100132771|LOC10013
NRN1
DKFZp566H0824
SCARNA11
MIRN21
MRLC2
FAM100B
RPL39L
MALAT1
LOC595101|LOC440354|LOC641298
PLOD2
MALAT1
RPS3
SCARNA9
RPS9
UBL5
CST3
H2AFZ
TMEM205
TIMM23
ZNF525|ZNF813
TAF13
HSPC152
TMEM59
KIF5B
HNRNPC
APP
SCARNA9
SEC61G
ND2|ND1
LOC440552|COX3
ARL6IP1
CALD1
SNORA61|C1orf79
ANP32A|LOC723972
IFRG15
MRPL51
SDCCAG1
NBPF16|NBPF15|NBPF8|LOC10013
NBPF15|NBPF10|NBPF9|NBPF14|LOC
NBPF16|NBPF15|LOC100132406|KIA
NBPF14|LOC100132406|KIAA1245|N
NBPF15|NBPF10|LOC100132406|KIA
NBPF16|NBPF15|LOC100132406|KIA
LOC100133459
SAP18
B2M
ANP32B
SSBP1
RTCD1
ATL3
PRSS23
HSP90AA1
RPS17
RPS17
CTSK
ND4L
CYTB
UFM1
COX1
RPL7A
COX6C
NPIP|LOC339047|LOC100132540|LC
TXNIP
COX7A2
NPIP|LOC440350|LOC339047|LOC10
A26C1A|A26C1B
PTMA|PTMAP7|PTMAP5
MYL6|MYL6B
A26C1B|A26C1A|FKSG30
LGR5
PDIA3P
LAPTM4A
SLC31A1
COX2
RPL24
CSDA
ND5
FTH1

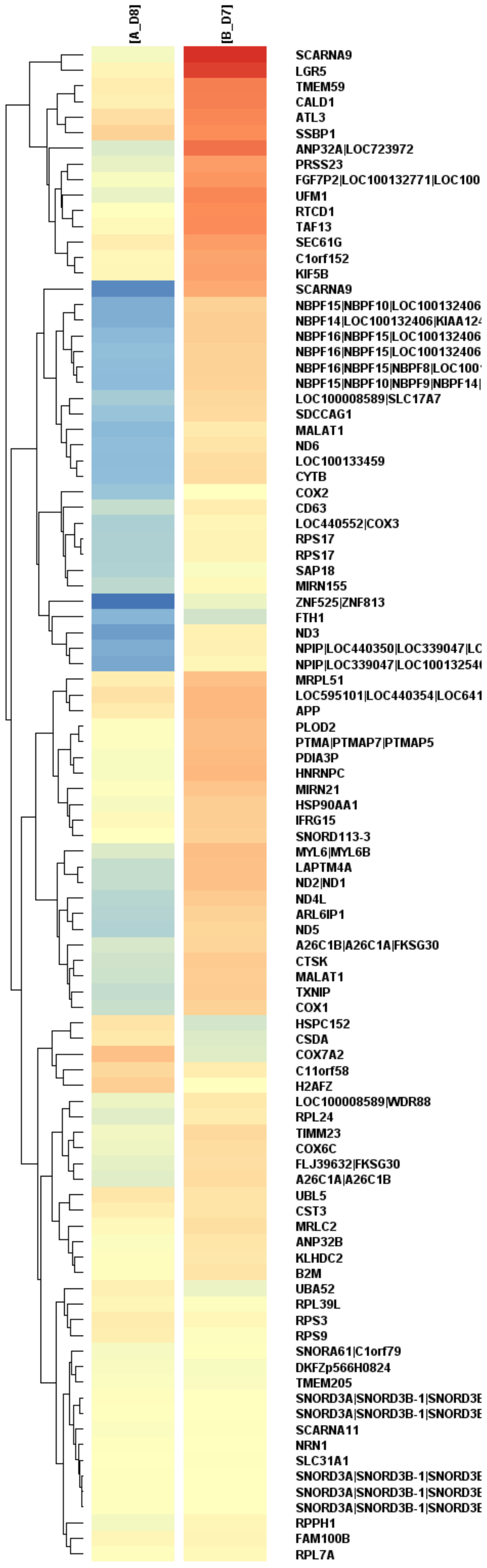
[A_D8]
[B_D7]
SCARNA9
LGR5
TMEM59
CALD1
ATL3
SSBP1
ANP32A|LOC723972
PRSS23
FGF7P2|LOC100132771|LOC100
UFM1
RTCD1
TAF13
SEC61G
C1orf152
KIF5B
SCARNA9
NBPF15|NBPF10|LOC100132406
NBPF14|LOC100132406|KIAA12
NBPF16|NBPF15|LOC100132406
NBPF16|NBPF15|LOC100132406
NBPF16|NBPF15|NBPF8|LOC100
NBPF15|NBPF10|NBPF9|NBPF14|
LOC100008589|SLC17A7
SDCCAG1
MALAT1
ND6
LOC100133459
CYTB
COX2
CD63
LOC440552|COX3
RPS17
RPS17
SAP18
MIRN155
ZNF525|ZNF813
FTH1
ND3
NPIP|LOC440350|LOC339047|LC
NPIP|LOC339047|LOC10013254
MRPL51
LOC595101|LOC440354|LOC641
APP
PLOD2
PTMA|PTMAP7|PTMAP5
PDIA3P
HNRNPC
MIRN21
HSP90AA1
IFRG15
SNORD113-3
MYL6|MYL6B
LAPTM4A
ND2|ND1
ND4L
ARL6IP1
ND5
A26C1B|A26C1A|FKSG30
CTSK
MALAT1
TXNIP
COX1
HSPC152
CSDA
COX7A2
C11orf58
H2AFZ
LOC100008589|WDR88
RPL24
TIMM23
COX6C
FLJ39632|FKSG30
A26C1A|A26C1B
UBL5
CST3
MRLC2
ANP32B
KLHDC2
B2M
UBA52
RPL39L
RPS3
RPS9
SNORA61|C1orf79
DKFZp566H0824
TMEM205
SNORD3A|SNORD3B-1|SNORD3
SNORD3A|SNORD3B-1|SNORD3
SCARNA11
NRN1
SLC31A1
SNORD3A|SNORD3B-1|SNORD3
SNORD3A|SNORD3B-1|SNORD3
SNORD3A|SNORD3B-1|SNORD3
RPPH1
FAM100B
RPL7A

Supplementary Figure S3. Functional category of the differentially expressed genes of α-SMA positive (“B” samples) and α-SMA negative (“A” samples) SSc fibroblasts.

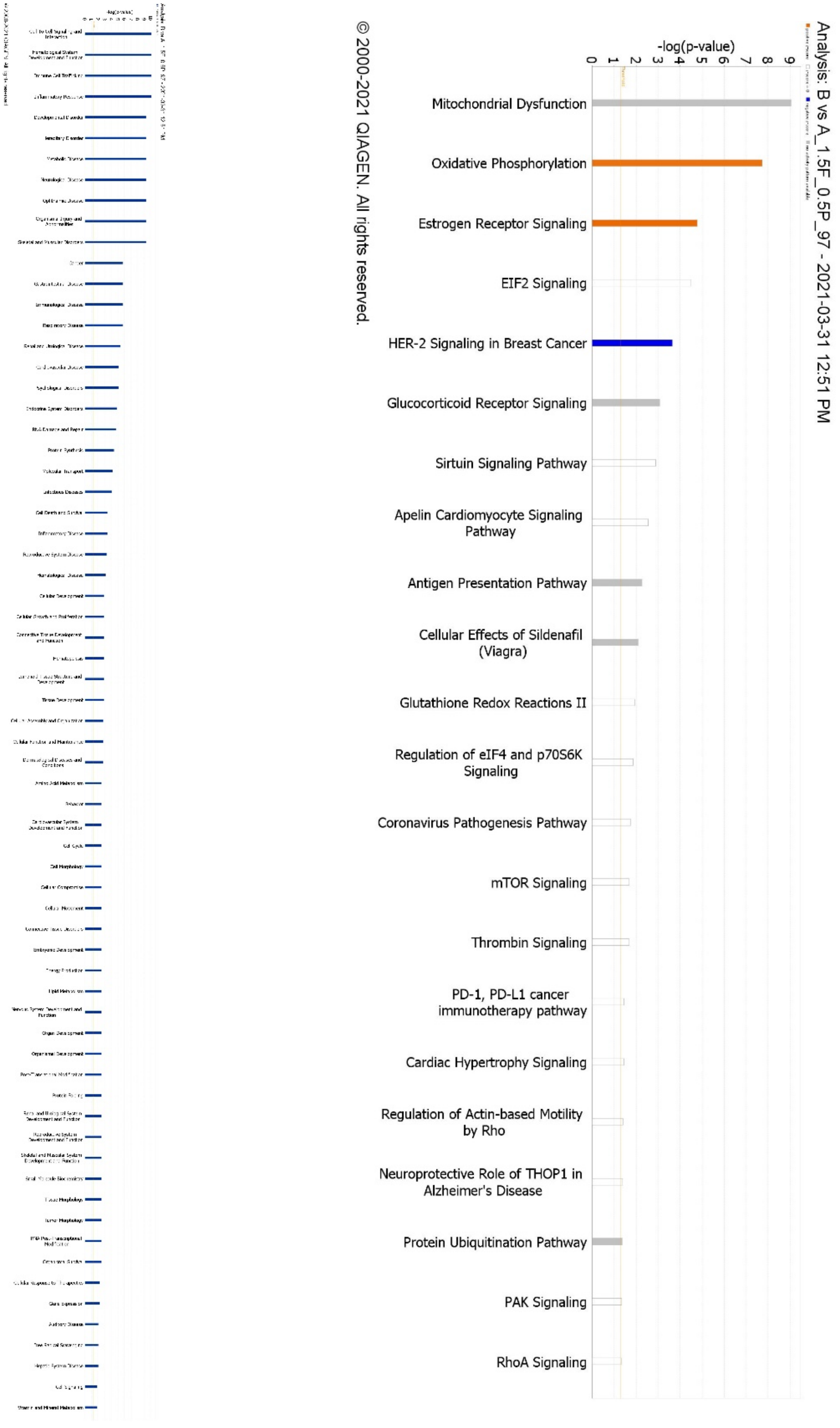

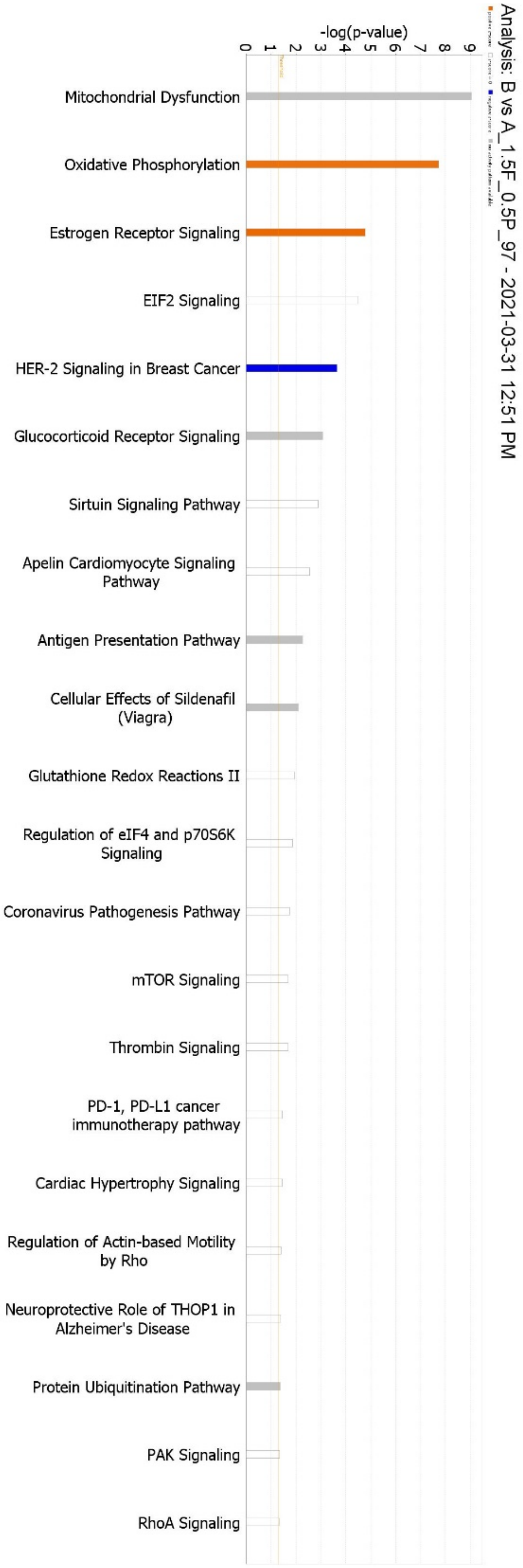

© 2000-2021 QIAGEN. All rights reserved.

Supplementary Table S1. Complete list of the genes and pathways identified by Ingenuity CanPath.

© 2000-2013 Ingenuity Systems, Inc. All rights reserved.

Regulation of Actin-based Motility by Rho	6.06E00	9.3E-02	MYL9,ACTR3,RND3,MYL6,ARPC2,ACTB,RHOA,ACTA2
RhoA Signaling	4.95E00	6.96E-02	MYL9,ACTR3,RND3,MYL6,ARPC2,ACTB,RHOA,ACTA2
RhoGDI Signaling	4.5E00	4.74E-02	MYL9,ACTR3,RND3,MYL6,ARPC2,ACTB,RHOA,ACTA2,GNG5
Epithelial Adherens Junction Signaling	4.24E00	5.56E-02	MYL9,ACTR3,LMO7,MYL6,ARPC2,ACTB,RHOA,ACTA2
Signaling by Rho Family GTPases	3.57E00	3.63E-02	MYL9,ACTR3,RND3,MYL6,ARPC2,ACTB,RHOA,ACTA2,GNG5
Integrin Signaling	3.35E00	3.9E-02	MYL9,ACTR3,RND3,ITGA11,ARPC2,ACTB,RHOA,ACTA2
Tight Junction Signaling	3.28E00	4.46E-02	MYL9,MYL6,RAB13,ACTB,RHOA,ACTA2,PRKAR1A
NRF2-mediated Oxidative Stress Response	2.89E00	3.74E-02	SOD1,MGST2,ACTB,ACTA2,UBE2K,DNAJA2,TXN
Actin Nucleation by ARP-WASP Complex	2.75E00	6.06E-02	ACTR3,RND3,ARPC2,RHOA
Mitochondrial Dysfunction	2.55E00	3.95E-02	NDUFA4,FIS1,MT-ND6,UQCRH,MT-CYB,COX6C
Remodeling of Epithelial Adherens Junctions	2.49E00	6.06E-02	ACTR3,ARPC2,ACTB,ACTA2
Actin Cytoskeleton Signaling	2.47E00	3.04E-02	MYL9,ACTR3,MYL6,ARPC2,ACTB,RHOA,ACTA2
Caveolar-mediated Endocytosis Signaling	2.35E00	4.94E-02	CD55,ITGA11,ACTB,ACTA2
Cellular Effects of Sildenafil (Viagra)	2.23E00	3.97E-02	MYL9,MYL6,ACTB,ACTA2,PRKAR1A
ILK Signaling	2.17E00	3.24E-02	MYL9,RND3,MYL6,ACTB,RHOA,ACTA2
Cdc42 Signaling	2.16E00	3.5E-02	MYL9,ACTR3,MYL6,ARPC2,EXOC5
IL-8 Signaling	2.15E00	3.12E-02	RAB11FIP2,MYL9,ANGPT1,RND3,RHOA,GNG5
Leukocyte Extravasation Signaling	2.04E00	3.06E-02	EDIL3,MYL6,ACTB,RHOA,ACTA2,TIMP2
Granzyme A Signaling	1.98E00	1.18E-01	SET,ANP32A
Fcγ Receptor-mediated Phagocytosis in Macrophages and Monocytes	1.96E00	4.21E-02	ACTR3,ARPC2,ACTB,ACTA2
Assembly of RNA Polymerase II Complex	1.96E00	5.45E-02	POLR2F,POLR2H,TAF13
CXCR4 Signaling	1.88E00	3.12E-02	MYL9,RND3,MYL6,RHOA,GNG5
Tec Kinase Signaling	1.85E00	2.86E-02	RND3,ACTB,RHOA,ACTA2,GNG5
Glioma Invasiveness Signaling	1.8E00	5.08E-02	RND3,RHOA,TIMP2
Cardiac Hypertrophy Signaling	1.79E00	2.62E-02	MYL9,RND3,MYL6,RHOA,GNG5,PRKAR1A
Choline Degradation I	1.73E00	5E-01	ALDH7A1
Androgen Signaling	1.72E00	3.05E-02	POLR2F,POLR2H,GNG5,PRKAR1A
Calcium Signaling	1.68E00	2.66E-02	MYL9,MYL6,ACTA2,TPM2,PRKAR1A
Ephrin Receptor Signaling	1.66E00	2.54E-02	ACTR3,ANGPT1,ARPC2,RHOA,GNG5
Estrogen Receptor Signaling	1.53E00	3.01E-02	POLR2F,H3F3A/H3F3B,POLR2H,TAF13
Thrombin Signaling	1.52E00	2.54E-02	MYL9,RND3,MYL6,RHOA,GNG5
Aryl Hydrocarbon Receptor Signaling	1.46E00	2.84E-02	HSP90B1,MGST2,NEDD8,ALDH7A1
Myo-inositol Biosynthesis	1.44E00	2.5E-01	IMPAD1

Hepatic Fibrosis / Hepatic Stellate Cell Activation	1.42E00	2.86E-02	MYL9,MYL6,ACTA2,TIMP2
Nucleotide Excision Repair Pathway	1.38E00	5.71E-02	POLR2F,POLR2H
Axonal Guidance Signaling	1.37E00	1.75E-02	MYL9,PAPPA,ACTR3,MYL6,ARPC2,RHOA,GNG5,PRKAR1A
Lysine Degradation II	1.34E00	2E-01	ALDH7A1
Lysine Degradation V	1.34E00	2E-01	ALDH7A1
Virus Entry via Endocytic Pathways	1.31E00	3.26E-02	CD55,ACTB,ACTA2
VEGF Signaling	1.31E00	3.16E-02	EIF1AX,ACTB,ACTA2
Mechanisms of Viral Exit from Host Cells	1.28E00	5E-02	ACTB,ACTA2
Thioredoxin Pathway	1.27E00	1.67E-01	TXN
Superoxide Radicals Degradation	1.27E00	1.67E-01	SOD1
Germ Cell-Sertoli Cell Junction Signaling	1.26E00	2.55E-02	RND3,ACTB,RHOA,ACTA2
Phospholipase C Signaling	1.22E00	2.05E-02	MYL9,RND3,MYL6,RHOA,GNG5
Paxillin Signaling	1.19E00	2.73E-02	ITGA11,ACTB,ACTA2
MSP-RON Signaling Pathway	1.19E00	4.26E-02	ACTB,ACTA2
Rac Signaling	1.15E00	2.56E-02	ACTR3,ARPC2,RHOA
CREB Signaling in Neurons	1.14E00	2.16E-02	POLR2F,POLR2H,GNG5,PRKAR1A
fMLP Signaling in Neutrophils	1.11E00	2.52E-02	ACTR3,ARPC2,GNG5
Agranulocyte Adhesion and Diapedesis	1.11E00	2.27E-02	MYL9,MYL6,ACTB,ACTA2
Semaphorin Signaling in Neurons	1.08E00	3.85E-02	RND3,RHOA
mTOR Signaling	1.05E00	2.12E-02	RND3,RHOA,RPS8,EIF4B
Clathrin-mediated Endocytosis Signaling	1.05E00	2.09E-02	ACTR3,ARPC2,ACTB,ACTA2
Gα12/13 Signaling	1.02E00	2.44E-02	MYL9,MYL6,RHOA
Glutamate Receptor Signaling	1.02E00	3.28E-02	SLC17A7,GNG5
Histamine Degradation	9.76E-01	8.33E-02	ALDH7A1
Fatty Acid α-oxidation	9.44E-01	7.69E-02	ALDH7A1
Hypoxia Signaling in the Cardiovascular System	9.38E-01	3.12E-02	HSP90B1,UBE2L3
Leukotriene Biosynthesis	9.13E-01	7.14E-02	MGST2
Vitamin-C Transport	9.13E-01	7.14E-02	TXN
Protein Kinase A Signaling	9.06E-01	1.61E-02	MYL9,H3F3A/H3F3B,MYL6,RHOA,GNG5,PRKAR1A
Agrin Interactions at Neuromuscular Junction	8.94E-01	2.99E-02	ACTB,ACTA2
The Visual Cycle	8.85E-01	6.25E-02	RDH10
Oxidative Ethanol Degradation III	8.85E-01	3.7E-02	ALDH7A1
Tryptophan Degradation X (Mammalian, via Tryptamine)	8.59E-01	6.25E-02	ALDH7A1
Putrescine Degradation III	8.59E-01	6.25E-02	ALDH7A1
Glutathione Redox Reactions I	8.35E-01	5.88E-02	MGST2
Ethanol Degradation IV	8.35E-01	5.88E-02	ALDH7A1
Ephrin B Signaling	8.34E-01	2.53E-02	RHOA,GNG5
BMP signaling pathway	8.25E-01	2.56E-02	FST,PRKAR1A

Glioblastoma Multiforme Signaling	8.21E-01	1.91E-02	IGF2,RND3,RHOA
Gαq Signaling	8.21E-01	1.91E-02	RND3,RHOA,GNG5
D-myo-inositol (1,4,5)-trisphosphate Degradation	8.12E-01	5.56E-02	IMPAD1
Gap Junction Signaling	7.84E-01	1.92E-02	ACTB,ACTA2,PRKAR1A
Colorectal Cancer Metastasis Signaling	7.74E-01	1.64E-02	RND3,RHOA,GNG5,PRKAR1A
Prostate Cancer Signaling	7.72E-01	2.2E-02	HSP90B1,PA2G4
DNA Methylation and Transcriptional Repression Signaling	7.7E-01	4.35E-02	SAP18
Dopamine Degradation	7.51E-01	4.76E-02	ALDH7A1
α-Adrenergic Signaling	7.31E-01	2.17E-02	GNG5,PRKAR1A
Bladder Cancer Signaling	7.23E-01	2.25E-02	PA2G4,THBS1
FAK Signaling	7.23E-01	2.04E-02	ACTB,ACTA2
Glutathione-mediated Detoxification	7.15E-01	4.35E-02	MGST2
G Beta Gamma Signaling	7.08E-01	2E-02	GNG5,PRKAR1A
PAK Signaling	7.08E-01	1.92E-02	MYL9,MYL6
Crosstalk between Dendritic Cells and Natural Killer Cells	7.01E-01	2.2E-02	ACTB,ACTA2
Superpathway of D-myo-inositol (1,4,5)-trisphosphate Metabolism	6.99E-01	4.17E-02	IMPAD1
IL-1 Signaling	6.86E-01	1.96E-02	GNG5,PRKAR1A
Protein Ubiquitination Pathway	6.8E-01	1.52E-02	USO1,HSP90B1,UBE2L3,USP53
HMGB1 Signaling	6.72E-01	2.06E-02	RND3,RHOA
EIF2 Signaling	6.71E-01	1.66E-02	EIF1AX,RPS8,RPL12
Sertoli Cell-Sertoli Cell Junction Signaling	6.66E-01	1.64E-02	ACTB,ACTA2,PRKAR1A
Glioma Signaling	6.65E-01	1.89E-02	IGF2,PA2G4
Glucocorticoid Receptor Signaling	6.64E-01	1.44E-02	HSP90B1,POLR2F,POLR2H,TAF13
Nitric Oxide Signaling in the Cardiovascular System	6.59E-01	1.96E-02	HSP90B1,PRKAR1A
Retinoate Biosynthesis I	6.26E-01	3.33E-02	RDH10
Sonic Hedgehog Signaling	6.26E-01	3.23E-02	PRKAR1A
Cholecystokinin/Gastrin-mediated Signaling	6.2E-01	1.98E-02	RND3,RHOA
Ethanol Degradation II	6.13E-01	3.33E-02	ALDH7A1
G Protein Signaling Mediated by Tubby	6.01E-01	2.7E-02	GNG5
Breast Cancer Regulation by Stathmin1	5.89E-01	1.52E-02	RHOA,GNG5,PRKAR1A
Inhibition of Angiogenesis by TSP1	5.89E-01	3.12E-02	THBS1
Noradrenaline and Adrenaline Degradation	5.89E-01	3.12E-02	ALDH7A1
Complement System	5.89E-01	3.03E-02	CD55
Sphingosine-1-phosphate Signaling	5.79E-01	1.74E-02	RND3,RHOA
Gαs Signaling	5.79E-01	1.74E-02	GNG5,PRKAR1A
Retinol Biosynthesis	5.78E-01	2.94E-02	RDH10
Hereditary Breast Cancer	5.57E-01	1.68E-02	POLR2F,POLR2H

Signaling			
CCR3 Signaling in Eosinophils	5.51E-01	1.65E-02	RHOA,GNG5
CD28 Signaling in T Helper Cells	5.51E-01	1.64E-02	ACTR3,ARPC2
Inhibition of Matrix Metalloproteases	5.26E-01	2.63E-02	TIMP2
P2Y Purigenic Receptor Signaling Pathway	5.26E-01	1.6E-02	GNG5,PRKAR1A
Netrin Signaling	5.17E-01	2.22E-02	PRKAR1A
Gαi Signaling	5.16E-01	1.56E-02	GNG5,PRKAR1A
Neuroprotective Role of THOP1 in Alzheimer's Disease	5.08E-01	2.38E-02	PRKAR1A
Transcriptional Regulatory Network in Embryonic Stem Cells	5.08E-01	2.5E-02	SET
eNOS Signaling	4.97E-01	1.55E-02	HSP90B1,PRKAR1A
Huntington's Disease Signaling	4.94E-01	1.31E-02	POLR2F,POLR2H,GNG5
Role of Oct4 in Mammalian Embryonic Stem Cell Pluripotency	4.74E-01	2.27E-02	PHB
Ovarian Cancer Signaling	4.66E-01	1.45E-02	PA2G4,PRKAR1A
Relaxin Signaling	4.61E-01	1.38E-02	GNG5,PRKAR1A
Cardiac β-adrenergic Signaling	4.61E-01	1.43E-02	GNG5,PRKAR1A
Ephrin A Signaling	4.43E-01	2E-02	RHOA
Amyloid Processing	4.29E-01	1.92E-02	PRKAR1A
Molecular Mechanisms of Cancer	4.26E-01	1.1E-02	RND3,PA2G4,RHOA,PRKAR1A
Phototransduction Pathway	4.23E-01	1.85E-02	PRKAR1A
Regulation of eIF4 and p70S6K Signaling	4.21E-01	1.27E-02	EIF1AX,RPS8
Serotonin Degradation	4.16E-01	1.92E-02	ALDH7A1
UVB-Induced MAPK Signaling	4.16E-01	1.85E-02	H3F3A/H3F3B
Cell Cycle: G1/S Checkpoint Regulation	3.74E-01	1.56E-02	PA2G4
Antiproliferative Role of Somatostatin Receptor 2	3.69E-01	1.56E-02	GNG5
Xenobiotic Metabolism Signaling	3.63E-01	1.12E-02	HSP90B1,MGST2,ALDH7A1
Mitotic Roles of Polo-Like Kinase	3.58E-01	1.56E-02	HSP90B1
PXR/RXR Activation	3.53E-01	1.56E-02	PRKAR1A
PPARα/RXRα Activation	3.47E-01	1.16E-02	HSP90B1,PRKAR1A
Angiopoietin Signaling	3.43E-01	1.41E-02	ANGPT1
Non-Small Cell Lung Cancer Signaling	3.43E-01	1.37E-02	PA2G4
CCR5 Signaling in Macrophages	3.33E-01	1.23E-02	GNG5
Melatonin Signaling	3.33E-01	1.47E-02	PRKAR1A
RAR Activation	3.33E-01	1.14E-02	RDH10,PRKAR1A
Growth Hormone Signaling	3.29E-01	1.41E-02	IGF2
Chemokine Signaling	3.29E-01	1.47E-02	RHOA
Macropinocytosis Signaling	3.29E-01	1.32E-02	RHOA

Small Cell Lung Cancer Signaling	3.15E-01	1.19E-02	PA2G4
PEDF Signaling	3.15E-01	1.3E-02	RHOA
Role of NFAT in Cardiac Hypertrophy	3.13E-01	1.07E-02	GNG5,PRKAR1A
Leptin Signaling in Obesity	3.07E-01	1.32E-02	PRKAR1A
Production of Nitric Oxide and Reactive Oxygen Species in Macrophages	3.05E-01	1.08E-02	RND3,RHOA
Dopamine Receptor Signaling	2.99E-01	1.32E-02	PRKAR1A
Cyclins and Cell Cycle Regulation	2.91E-01	1.15E-02	PA2G4
ERK/MAPK Signaling	2.9E-01	1.01E-02	H3F3A/H3F3B,PRKAR1A
Melanocyte Development and Pigmentation Signaling	2.65E-01	1.15E-02	PRKAR1A
Neuregulin Signaling	2.59E-01	1.05E-02	HSP90B1
CDK5 Signaling	2.55E-01	1.12E-02	PRKAR1A
PPAR Signaling	2.46E-01	1E-02	HSP90B1
Chronic Myeloid Leukemia Signaling	2.4E-01	9.8E-03	PA2G4
Systemic Lupus Erythematosus Signaling	2.38E-01	8.66E-03	ZCRB1,HNRNPC
p53 Signaling	2.37E-01	1.05E-02	THBS1
SAPK/JNK Signaling	2.37E-01	1E-02	GNG5
LPS/IL-1 Mediated Inhibition of RXR Function	2.36E-01	9.01E-03	MGST2,ALDH7A1
Antioxidant Action of Vitamin C	2.34E-01	1.02E-02	TXN
Telomerase Signaling	2.28E-01	1.01E-02	HSP90B1
Amyotrophic Lateral Sclerosis Signaling	2.25E-01	1E-02	SOD1
IGF-1 Signaling	2.25E-01	9.8E-03	PRKAR1A
Neuropathic Pain Signaling In Dorsal Horn Neurons	2.2E-01	9.8E-03	PRKAR1A
Pancreatic Adenocarcinoma Signaling	2.02E-01	8.7E-03	PA2G4
NGF Signaling	2.02E-01	9.01E-03	RHOA
Corticotropin Releasing Hormone Signaling	2E-01	8.77E-03	PRKAR1A
Renin-Angiotensin Signaling	1.97E-01	8.85E-03	PRKAR1A

Supplementary Figure S4. List of the RNA samples used in the microarray experiments with their concentrations, the 260/280 ratio for demonstration of their high purity and the nanodrop analysis.

SSc Patient	SMA	Nanodrop ID	ng/microl	260/280
1	+	1	425.8	1.85
1	-	2	453	1.81
2	+	3	188.9	1.95
2	-	4	150	1.94
3	+	5	218.1	1.94
3	-	6	202.7	1.93
4	+	7	150.6	1.92
4	-	8	186.9	1.89

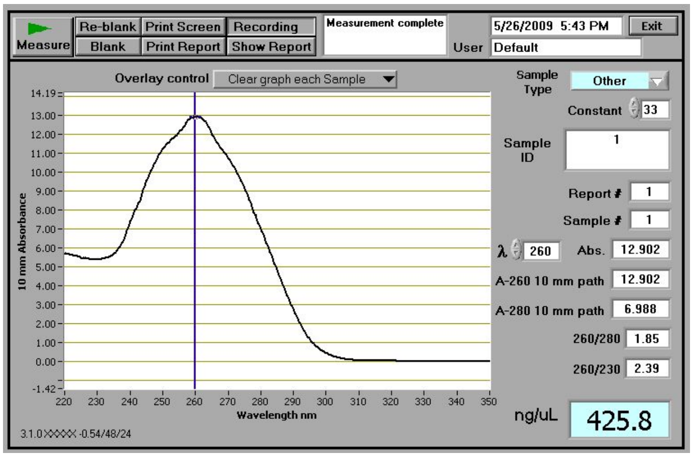

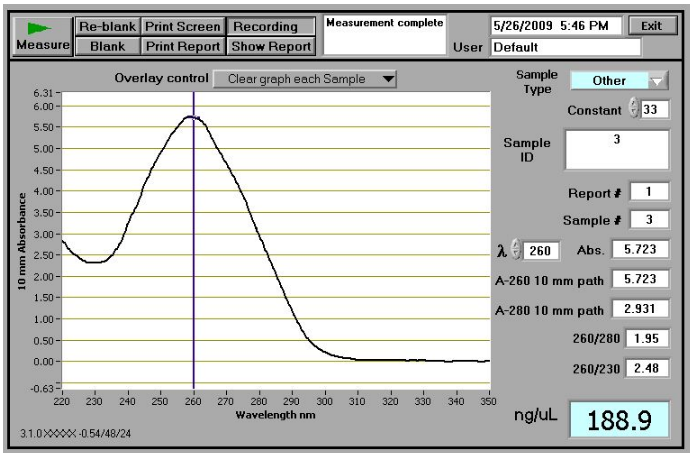

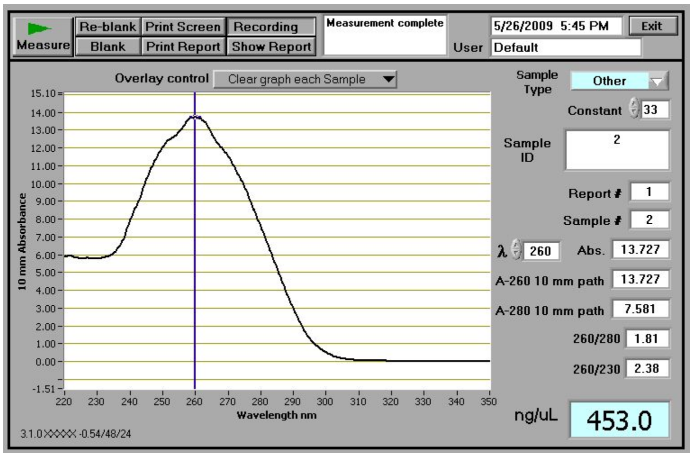

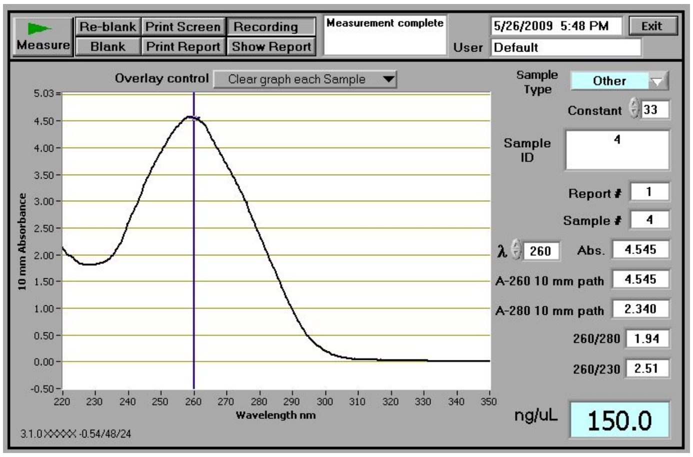

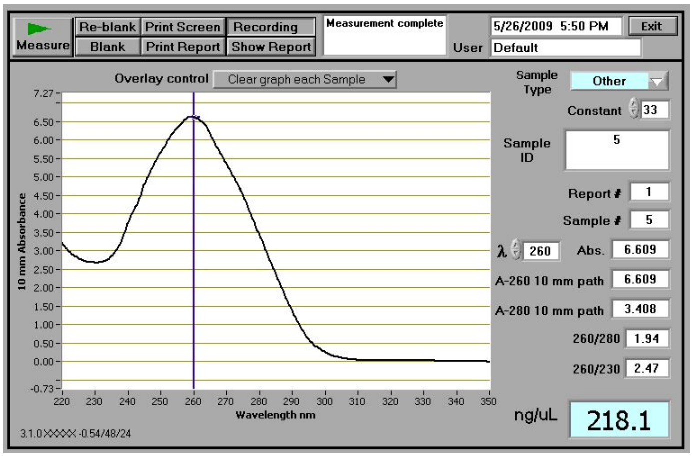
Measure
Re-blank
Print Screen
Recording
Blank
Print Report
Show Report
Measurement complete
5/26/2009 5:50 PM
Exit
User
Default
Overlay control
Clear graph each Sample
Sample Type
Other
Constant
33
Sample ID
5
Report #
1
Sample #
5
λ
260
Abs.
6.609
A-260 10 mm path
6.609
A-280 10 mm path
3.408
260/280
1.94
260/230
2.47
ng/uL
218.1
10 mm Absorbance
Wavelength nm
3.1.0 XXXXX -0.54/48/24

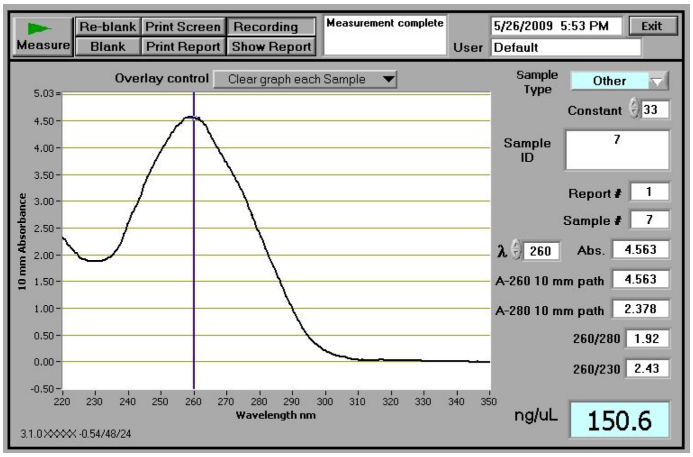
Measure
Re-blank
Print Screen
Recording
Blank
Print Report
Show Report
Measurement complete
5/26/2009 5:53 PM
Exit
User
Default
Overlay control
Clear graph each Sample
Sample Type
Other
Constant
33
Sample ID
7
Report #
1
Sample #
7
λ
260
Abs.
4.563
A-260 10 mm path
4.563
A-280 10 mm path
2.378
260/280
1.92
260/230
2.43
ng/uL
150.6
10 mm Absorbance
Wavelength nm
3.1.0 XXXXX -0.54/48/24

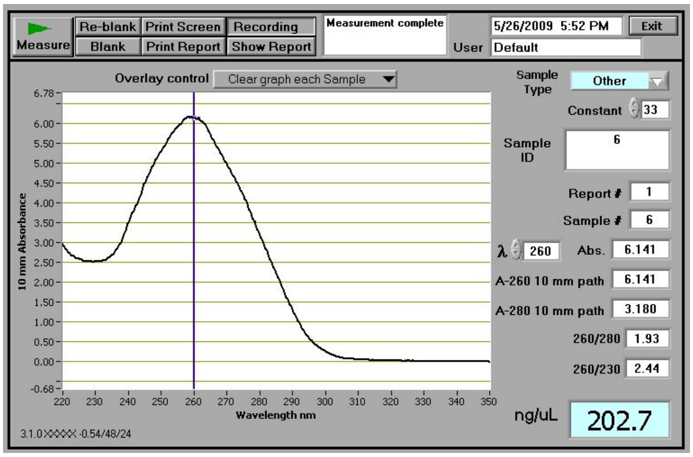
Measure
Re-blank
Print Screen
Recording
Blank
Print Report
Show Report
Measurement complete
5/26/2009 5:52 PM
Exit
User
Default
Overlay control
Clear graph each Sample
Sample Type
Other
Constant
33
Sample ID
6
Report #
1
Sample #
6
λ
260
Abs.
6.141
A-260 10 mm path
6.141
A-280 10 mm path
3.180
260/280
1.93
260/230
2.44
ng/uL
202.7
10 mm Absorbance
Wavelength nm
3.1.0 XXXXX -0.54/48/24

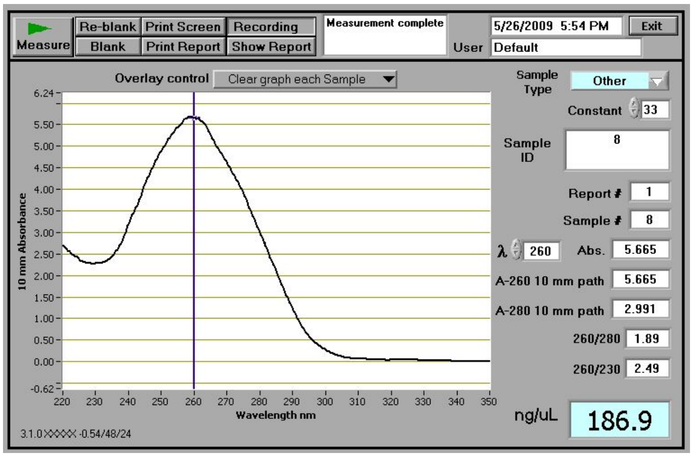
Measure
Re-blank
Print Screen
Recording
Blank
Print Report
Show Report
Measurement complete
5/26/2009 5:54 PM
Exit
User
Default
Overlay control
Clear graph each Sample
Sample Type
Other
Constant
33
Sample ID
8
Report #
1
Sample #
8
λ
260
Abs.
5.665
A-260 10 mm path
5.665
A-280 10 mm path
2.991
260/280
1.89
260/230
2.49
ng/uL
186.9
10 mm Absorbance
Wavelength nm
3.1.0 XXXXX -0.54/48/24

Supplementary Figure S5. Full blots of Western Blot included in Figure 3.

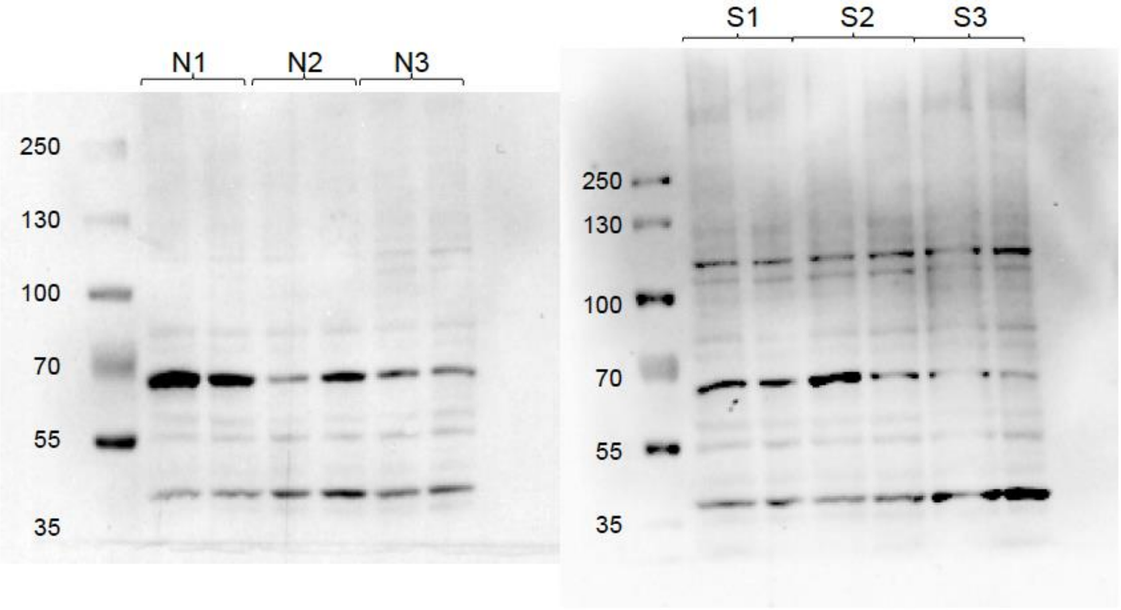

Supplement: Supplementary file 2 — Supplementary Information 2. [file 41598_2021_99292_MOESM2_ESM.pdf]
